# Optical Detection Method for High Aspect Ratio Microstructures

**DOI:** 10.3390/mi11030296

**Published:** 2020-03-11

**Authors:** Wenbin Wei, Shuangyue Hou, Zhao Wu, Yue Hu, Yi Wang, Lijuan Chen, Ying Xiong, Yangchao Tian, Gang Liu

**Affiliations:** National Synchrotron Radiation Laboratory, University of Science and Technology of China, Hefei 230029, China; wwb1992@mail.ustc.edu.cn (W.W.); hsy58@mail.ustc.edu.cn (S.H.); wuzhao@ustc.edu.cn (Z.W.); ycyzhy12@mail.ustc.edu.cn (Y.H.); ritawang@mail.ustc.edu.cn (Y.W.); chlj@mail.ustc.edu.cn (L.C.); xywch@ustc.edu.cn (Y.X.); ychtian@ustc.edu.cn (Y.T.)

**Keywords:** optical detection, UV-LIGA, high aspect ratio, microstructures

## Abstract

High aspect ratio microstructures (HARMS) are of great importance for many application fields. Many defects are generated during the fabrication processes, especially in line microstructures, and it is necessary to examine the quality of the structures after each process. However, there is no suitable efficient nondestructive detection method to monitor microstructures during the fabrication processes. In this paper, an optical detection method capable of detecting the structures by analyzing the reflection of light on the line HARMS is proposed. According to the image of reflected visible light, this method can determine whether there are defects in structures, so as to realize efficient detection. Preliminary simulations and experiments have been performed to confirm the feasibility and validity of the proposed method for detecting line microstructures. This method is expected to obtain more information about microstructures by further optimizing system parameters.

## 1. Introduction

High aspect ratio microstructures (HARMS) are widely used in many fields, such as optics [1,2,3] and MEMS (Microelectromechanical systems) [4,5,6,7,8]. UV-LIGA (Ultraviolet-Lithographie, Galvanoformung, Abformung) is considered as a powerful technique to fabricate HARMS with the linewidth of greater than 10 microns and the height of hundreds of microns [9,10,11,12]. However, during the UV lithography, development and electroplating processes, owing to the diffraction effect and the limitations of the mechanical strength of photoresist, the pattern is easily deformed, such as trapezium, distortion, tilt, and collapse [13,14,15]. These deformations could influence the performance of microstructures.

There are many methods that have been developed to prevent or reduce pattern collapse in technical processes [14,15,16,17,18,19], but it is still a challenge to keep HARMS upright without collapse. In order to ensure the microstructures meet the design requirements, it’s vital to monitor the quality of the structures after each process without bringing additional defects to the pattern or interrupting the fabrication processes. In general, height, linewidth and the shape of the sidewall of the microstructure need to be detected. For HARMS, the shape of the sidewall is the most important feature to characterize the quality of the structure. Almost all deformations of the microstructure are reflected in the changes of the shape of the sidewalls. The slight deformation of the sidewalls can have a great influence on HARMS, and the detection of the sidewalls is of great significance.

For the detection of HARMS, the current methods include optical microscopy, white light interferometry [20,21], scanning electron microscopy (SEM) [22,23], optical scatterometry [24,25], small-angle X-ray scattering metrology [26,27], atomic force microscopy [25,28], and stylus profiler [25,29]. It’s a challenge for these methods to quickly detect such microstructures with the height of hundreds of microns during the fabrication processes. Optical microscopy and white light interferometry can measure the linewidth at the top of the microstructure, but the measurement of the shape of the sidewall is difficult for them. SEM can measure microstructures with high accuracy. However, microstructures are usually in a variety of environments during the fabrication processes and are not suitable for SEM measurement. Optical scatterometry and small-angle X-ray scattering metrology can be used to detect nanostructures with sub-nanometer precision, rather than the microstructures of ten to hundreds of microns. As for atomic force microscopy, and stylus profiler, it is difficult to insert a scanning probe into the bottom, which is several hundred microns deep. 

In general, it seems inappropriate for these methods to detect microstructures with the height of several hundred microns during the fabrication processes. Thus, a high-efficiency detection method is required to detect the HARMS.

A novel optical detection method for HARMS is presented here. By analyzing the reflection of the light on the microstructures, it can quickly and nondestructively detect whether the structure is distorted or tilted, and quantitatively calculate the position and degree of the defect. The simulated results, as well as experimental results, verify the feasibility of the method.

## 2. Materials and Methods 

Most microstructures are composed of line segments, arcs and other shapes. Line patterns are more prone to deform due to their larger sidewalls and lower mechanical strength, and the deformation is not easy to observe from the surface. It is more necessary to detect line microstructures. Therefore, the detection method of the line microstructures was studied. To better elucidate the principle of the method and show the detection results more intuitively, periodic microstructures consisting of parallel segments are analyzed here.

**Figure 1:**
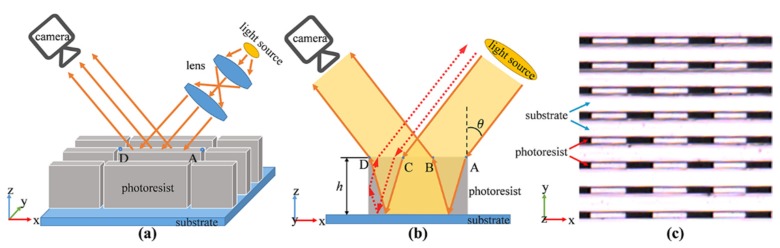
(**a**) Schematic diagram of the optical detection system; (**b**) schematic diagram of light propagation in photoresist structure; (**c**) image of the microstructures observed by the optical detection method.

The optical detection method uses a parallel obliquely incident beam along the direction of the microstructure’s groove and detects the intensity distribution of the reflected light to analyze the structures. The optical detection system is shown in Figure 1a, including an LED light source, a lens system and a CCD (charge-coupled device) camera array with a long working distance objective lens.

By observing the reflected light, it can be found that one end of the line segment structure is bright and the other end is dark, and the bright and dark phenomenon of each structure has a good consistency, as shown in Figure 1c. The optical detection method can determine whether there is deformation by analyzing the light and dark phenomenon of a single structure without referring to other structures, even if there is only one single structure.

To explain this phenomenon, it is necessary to analyze the propagation of light within a single photoresist structure. Figure 1b is the section diagram of the microstructures along the groove direction, showing the propagation of light in a photoresist structure. The light enters the photoresist structure from the rightmost point A, reaches the surface of the substrate and then reflects, and finally exits from point B. When the incident light reaches point C, it will exit from the leftmost point D. If the incident light reaches between the CD regions, it will strike on the sidewall. Because the refractive index of the photoresist is higher than the outside regions, the light will be totally internally reflected by the sidewalls within a certain range of the incident angles, and exits from the direction which is exactly opposite to the incident direction as the dotted arrow shows. Since there is no light emitted from the AB region, only light emitted from the BD region can be observed on the CCD camera. The length of BD and the height of the photoresist structure can be calculated by the following equations respectively:

(1) $$L_{BD}=L_{AD}-L_{AB}=L_{AD}-2h\frac{n_{1}\sin\theta }{\sqrt{n_{2}^{2}-n_{1}^{2}\sin^{2}\theta }},$$

(2) $$h=\frac{L_{AB}\sqrt{n_{2}^{2}-n_{1}^{2}\sin^{2}\theta }}{2n_{1}\sin\theta },$$

where *h* is the height of the photoresist structure, *n_1_* is the refractive index of the medium above the photoresist, *n_2_* is the refractive index of photoresist, *θ* is the incident angle of light, *L_AB_*, *L_BD_* and *L_AD_* are the lengths of AB, BD and AD regions, respectively. The medium above the photoresist could be air or liquid, and *n_1_* is the refractive index of air or liquid.

Ideally, the sidewalls of the structures are steep, so it should not affect the distribution of the outgoing lights. However, if the photoresist structures are tilted and distorted during the fabrication, the sidewalls are no longer steep, and the exiting light will be affected by the photoresist structures, so the exiting light should be redistributed, which is different from the ideal case.

**Figure 2:**
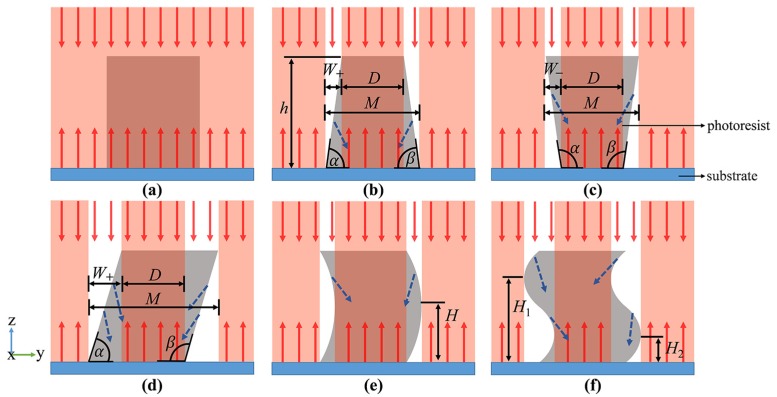
Schematic diagram of light reflected by structures with different shapes. (**a**) Standard rectangular structure; (**b**) trapezoidal structure with acute base angle; (**c**) trapezoidal structure with obtuse base angle; (**d**) tilted structure; (**e**) C-shape distorted structure; (**f**) S-shape distorted structure.

In order to analyze the influence of the structure shape on the exiting light, it is necessary to observe the reflection of light from the cross-section of the structures. Figure 2 shows the reflection of light in structures with different shapes, (a) the ideal structure with vertical sidewall; (b) trapezoidal structure with acute angle on base edge; (c) trapezoidal structure with obtuse angle on base edge; (d) tilted structure with one acute and one obtuse angle on base edge; (e) structure with C-shape distortion; and (f) structure with S-shape distortion. It can be seen from Figure 2 that when the sidewall of the structure is tilted or twisted, the incident light is refracted or reflected away from the original direction and cannot be reflected vertically by the substrate, shown as blue dotted arrows. Only the light in the red region can be reflected vertically into the CCD camera. Thus, these red regions are bright and the rest are dark in the captured image. According to the analysis in Figure 2, it can be seen that the structure can be monitored by analyzing the distribution of the reflected light, thereby realizing fast and straightforward detection.

The distribution of the light is related to the shape of the photoresist structure. Conversely, the structural parameters can be derived by the width of the dark regions. The base angle *α* of the structures shown in Figure 2b–d can be calculated by the following equations:

(3) $$\alpha =\{\begin{array}{c} \arctan\left(\frac{h}{W_{+}}\right)\\ 180{}^{\circ}-\arctan\left(\frac{h}{W_{-}}\right) \end{array},$$

where *h* is the height of the structure, *W* is the projected width of the sidewall, subscripts + and − indicate that the dark region appears outside and inside the top area of the photoresist, respectively. Similarly, the base angle *β* can also be calculated using Equation (3).

**Figure 3:**
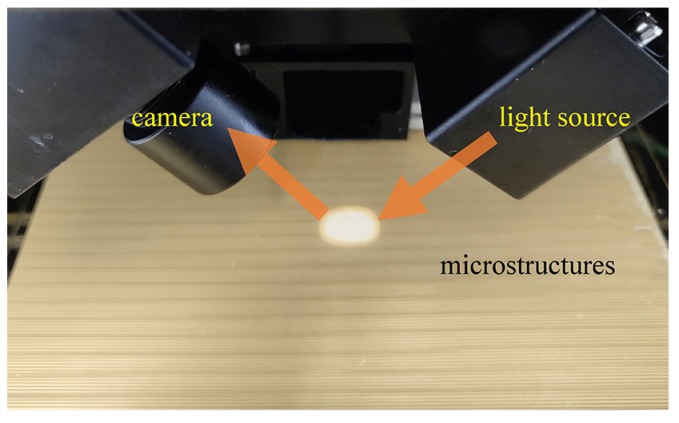
The setup of the optical detection system.

In order to validate the feasibility and validity of the presented optical detection method, a series of simulation-based and experimental studies were performed. The optical simulations were analyzed by the ray optics module of commercial COMSOL Multiphysics software. The intensity distributions of the lights reflected by microstructures with different shapes were simulated. In the experimental studies, a series of microstructures, which were fabricated by UV exposure and development, were detected by the proposed method. The dose for the 250 µm layer of SU-8 photoresist was about 360 mJ/cm^2^ in UV exposure [30]. After an appropriate development, the microstructures were fabricated. The optical detection setup is shown in Figure 3, an expanded collimated beam (diameter = 10 mm) from an LED light source was formed using a pair of convex lenses. The obliquely incident beam was along the direction of the microstructures groove and then reflected into the camera. The incident angle of light in this setup is 40°.

## 3. Results and Discussion

### 3.1. Simulated Results

**Figure 4:**
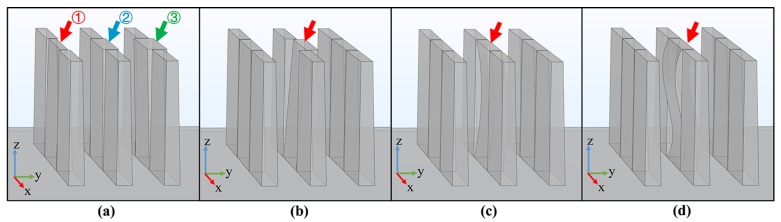
3D geometrical models of the structures with different shapes. (**a**) Trapezoidal structure with different base angles: ① 88°, ② 90°, and ③ 92°; (**b**) tilted structure; (**c**) C-shape distorted structure; (**d**) S-shape distorted structure.

The 3D geometrical models of the structures, which contained different shapes, are shown in Figure 4. The height of the structure was 300 μm, the length was 440 μm, the period was 120 μm, and the linewidth was 40 μm. The material of the photoresist was defined as exposed SU-8 2100 negative photoresist, which has a refractive index of 1.59 when the wavelength of light is 600 nm [30]. The area above the structure was defined as air with a refractive index of 1. The material of the substrate was defined as a polished silicon wafer, and light can be specularly reflected on its surface. The incident light was set to a parallel beam and along the direction of the structure groove with the incident angle of 40°. A camera was used to detect the distribution of the reflected light. In Figure 4a, the base angles of the structure indicated by arrows ①, ②, and ③ are 88°, 90°, and 92° respectively. The structures pointed by the arrows in Figure 4b–d are tilted, C-shape distortion and S-shape distortion, respectively. It should be mentioned that considering the diffraction in UV exposure, it is difficult to achieve 90° for the base angle of the photoresist structure. Generally, a base angle of 89° is excellent in actual processing. Therefore, the remaining structures as standard microstructures for reference in Figure 4 are all trapezoidal sidewalls with a base angle of 89°.

**Figure 5:**
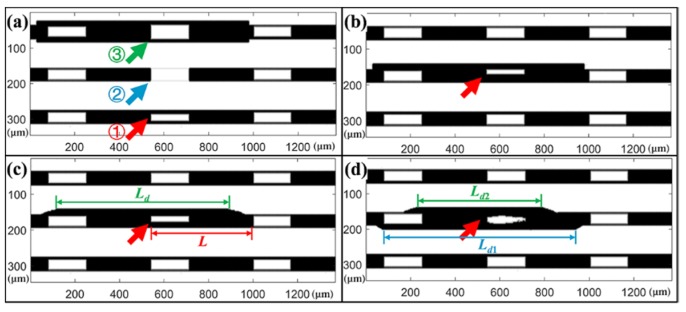
The simulated results of light reflected by structures with different shapes. (**a**) Trapezoidal structure with different base angles: ① 88°, ② 90°, and ③ 92°; (**b**) tilted structure; (**c**) C-shape distorted structure; (**d**) S-shape distorted structure.

The simulated results of the captured picture are shown in Figure 5, where the structures are consistent with Figure 5a–d. In Figure 5a, the sidewalls of the structure indicated by the arrow ① are trapezoidal with a base angle of 88°. Since the tilted sidewalls cannot reflect light vertically, dark stripes appear in these regions. Due to the difference of the base angle, the widths of these dark stripes are different. The dark stripes indicated by arrow ① are significantly wider than others. The base angle of the structure indicated by arrow ② is 90°, the steep sidewall does not affect the reflection of light, so there is no dark stripe. When the base angle is 92°, the top of the structure is wider than the bright region, as shown by arrow ③. In Figure 5b, the structure indicated by the arrow is tilted. It can be seen that the bright area in the structure is narrowed. The light reflected from the substrate also changed, darkening in the area near the inclined side of the structure. In Figure 5c, a C-shaped distortion appears in the structure indicated by the arrow. The light emitted from the inside of the structure is thinned, which is similar to Figure 5b. The distortion also affects the light reflected from the substrate. The protruding sidewall blocks the light from the substrate, causing the area near the distortion side of the structure to darken. The length of the dark area *L_d_* can be described as:

(4) $$L_{d}=L+2H\tan\theta ,$$

where *L* is the length of the photoresist structure, *θ* is the incident angle of light, *H* is the height of the position where the structure is deformed as shown in Figure 2d. Then *H* can be expressed as:

(5) $$H=\frac{L_{d}-L}{2\tan\theta }.$$

In Figure 5d, the S-shaped distortion of the structure is indicated by the arrow. As can be seen from the figure, the light emitted from the inside of the structure is narrowed and in an irregular shape. The S-shaped distortion of the structure causes regions on both sides of the structure to darken. As is mentioned before in Equation (5), the longer *L_d_*, the higher *H*. So it can be easily concluded that the position of the distortion *H*_1_ is higher than *H*_2_ as the length of the dark regions *L_d_*_1_ is longer than *L_d_*_2_, which is consistent with the models in Figure 2f and Figure 4d. The degree and direction of distortion can be obtained by the height of the distortion position and the width of the dark region. Based on this information, the exact shape of the structure can be analyzed.

The simulated results in Figure 5 are in good agreement with the analysis in Figure 2. From the simulated results, one can easily find that the reflection of the light of the structures with defects is significantly different from the standard structure. Thus, the optical detection method is valid to be used in quickly determining the defects inside the microstructures. For the photoresist structures, it can quantitatively calculate the position and degree of the defect and analyze the shape of the structures.

### 3.2. Experimental Results

**Figure 6:**
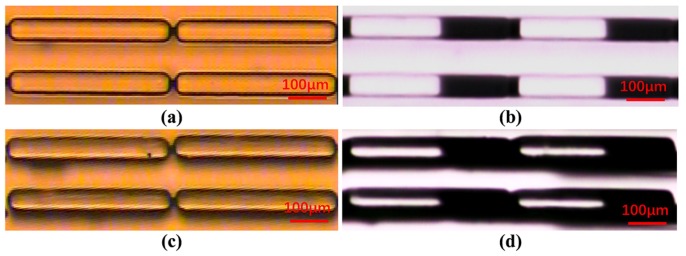
(**a**) Optical microscopic image of the normal structures; (**b**) optical detection image of the normal structures; (**c**) optical microscopic image of the tilted structures; (**d**) optical detection image of the tilted structures.

The proposed method was used to detect the fabricated microstructures and the results were compared with optical microscopy. The optical microscopic images of normal and tilted structures are shown in Figure 6a,c, respectively. The optical detection images of the corresponding structures are shown in Figure 6b,d. It can be seen that there is only a slight difference between the tilted and normal structure in the optical microscopic images, and it is difficult to determine which kinds of defects exist in the microstructures. However, they are quite different in the optical detection images. It can be seen that there are obvious dark stripes on both sides of the tilted structures, and the bright stripes in these tilted photoresist structures are significantly thinner and darker than the normal structures.

**Figure 7:**
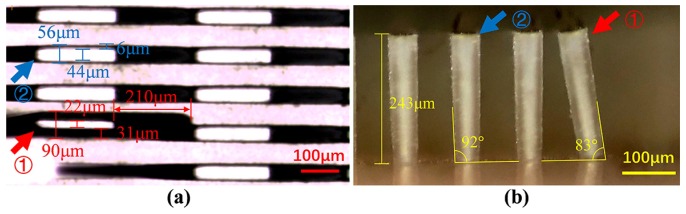
(**a**) Optical detection image of the tilted structure, and (**b**) microscopic side view of the same structures.

The optical detection image of the tilted structures and the optical microscopic image of the cross-section are shown in Figure 7. The length of the dark region is 210 μm, so that the height of the structure should be 238 μm according to Equation (2). The width of the dark area indicated by arrow ① is 31 μm, and the base angle of the structures should be 82.6° using Equation (3). The width of the dark area indicated by arrow ② is 6 μm, and the base angle of the structures should be 92.4°. Through the microscopic side view in Figure 7b, the height of the structure can be measured to be 243 μm, the two base angles are 83° and 92°. The calculated results of the optical detection method are in good agreement with the measured results of the cross-section, which indicates that the proposed method can quantitatively detect the structure without destroying it. The detection sensitivity of the optical detection method is limited by the spatial resolution of the CCD. In our detection system, the effective pixel size is 1.67 μm, and for a microstructure with a height of 250 μm, the angular sensitivity which indicates the minimum variation of the sidewall angle that can be detected is about 0.4°. The angular sensitivity can be optimized to 0.1° by using a higher resolution CCD and a higher magnification objective lens.

**Figure 8:**
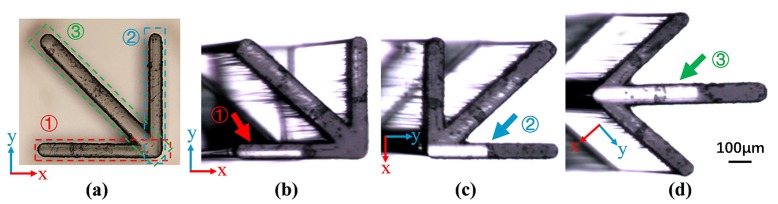
(**a**) Optical microscopic image of microstructures consist of three line segments; (**b**) optical detection image of the line segment ①; (**c**) optical detection image of the line segment ②; (**d**) optical detection image of the line segment ③.

Except for these periodic parallel segments, the optical detection method is also applicable to microstructures composed of line segments. A defective microstructure consisting of three line segments was fabricated by inclined exposure. Figure 8a is the optical microscopic image of the microstructures. During the UV exposure, the wafer was titled about 4° around the ***X***-axis instead of perpendicular to the UV rays. Since the angle between the UV rays and the ***Y***-axis is 86°, line segments ① and ③ would tilt to different degrees along the ***Y***-axis direction, while line segment ② would not tilt because it is parallel to the ***Y***-axis. The optical detection images of the microstructure are shown in Figure 8b–d. These line segments, which look like lamellas, can be detected by rotating the observation angle. As shown in Figure 8b,d, the bright stripes indicated by arrows ① and ③ become thinner and significantly closer to one side, indicating that the structures have been tilted. In Figure 8c, the bright stripe pointed by arrow ② is intact, which proves that the structure has not been deformed. After calculation, the tilt angle of line segments ① and ③ in the directions perpendicular to the sidewalls are 3.7° and 2.8°, respectively. This is consistent with the expected results of the experiment.

Based on the above experimental results, it can be seen that the optical detection method can effectively detect the deformation of HARMS and judge whether the structures meet the design requirements. Moreover, this method can be used to quantitatively calculate the position and degree of defects in the photoresist structures. It should be mentioned that the optical detection method is specifically for the microstructures which are composed of line segments, and is not suitable for other shapes, such as arcs and dots.

An important principle for the detection of microstructures is that any detection method for microstructures should not deform the microstructures during the detection process. Particularly, the photoresist microstructures after UV lithography should be carefully examined in the liquid environment to prevent the pattern from collapsing due to capillary forces, and then the faultless photoresist microstructures can be transferred to the further process [16]. Thanks to the non-destructive and non-contact properties of optics, the proposed method is expected to realize the in situ detection of microstructure in liquid environments, which is exactly the next research work to be performed. The appropriate incident angle should be determined based on the length of the microstructure, the refractive index of the microstructure and the environment. At present, the detection system is preliminary, many components are fixed and have no degrees of freedom. In order to be able to adapt to most cases of our samples, the incident angle of the preliminary detection system is fixed at 40°. The detection system will be updated in the subsequent research plan and the angle of incidence and reflection can be adjusted. It may be possible to obtain more information about the microstructures by capturing reflected light at different angles. Although the proposed method may not as accurate as some methods, it is an optional detection method that is fast and convenient.

## 4. Conclusions

In this work, an optical detection method has been proposed to detect the line HARMS and calculate its structure parameters. According to the image of reflected visible light, the proposed method can determine whether there are defects in structures, it offers a quick and effective detection tool for the microstructures. By analyzing the reflection of light on the HARMS, one can quantitatively calculate the position and degree of defects in the photoresist structures. The proposed method is applicable for the detection of HARMS composed of lamellas. The simulations and experiments demonstrate the feasibility of this optical method in HARMS detection. Meanwhile, owing to the obvious features and high contrast of the optical detection image, automatically analyzing the images could be achieved. Combined with automatic scanning, this method can be used to detect large area microstructures and then greatly improve the efficiency of detection. The proposed method has the potential to be applied in real-time monitoring of the fabrication processes in the near future.
